# Traits and climate are associated with first flowering day in herbaceous species along elevational gradients

**DOI:** 10.1002/ece3.3720

**Published:** 2017-12-20

**Authors:** Solveig Franziska Bucher, Patrizia König, Annette Menzel, Mirco Migliavacca, Jörg Ewald, Christine Römermann

**Affiliations:** ^1^ Department of Plant Biodiversity Institute of Ecology and Evolution Friedrich Schiller University Jena Jena Germany; ^2^ German Centre for Integrative Biodiversity Research (iDiv) Halle‐Jena‐Leipzig Leipzig Germany; ^3^ Department of Ecology and Ecosystem Management Technische Universität München Freising Germany; ^4^ Institute for Advanced Study Technische Universität München Garching Germany; ^5^ Biosphere‐Atmosphere Interactions and Experimentation Max Planck Institute for Biogeochemistry Jena Germany; ^6^ Department of Forest Science and Forestry Weihenstephan‐Triesdorf University of Applied Sciences Freising Germany

**Keywords:** altitude, carbon isotope discrimination (Δ^13^C), leaf nutrients, phenology, specific leaf area, stomatal pore area index

## Abstract

Phenological responses to changing temperatures are known as “fingerprints of climate change,” yet these reactions are highly species specific. To assess whether different plant characteristics are related to these species‐specific responses in flowering phenology, we observed the first flowering day (FFD) of ten herbaceous species along two elevational gradients, representing temperature gradients. On the same populations, we measured traits being associated with (1) plant performance (specific leaf area), (2) leaf biochemistry (leaf C, N, P, K, and Mg content), and (3) water‐use efficiency (stomatal pore area index and stable carbon isotopes concentration). We found that as elevation increased, FFD was delayed for all species with a highly species‐specific rate. Populations at higher elevations needed less temperature accumulation to start flowering than populations of the same species at lower elevations. Surprisingly, traits explained a higher proportion of variance in the phenological data than elevation. Earlier flowering was associated with higher water‐use efficiency, higher leaf C, and lower leaf P content. In addition to that, the intensity of shifts in FFD was related to leaf N and K. These results propose that traits have a high potential in explaining phenological variations, which even surpassed the effect of temperature changes in our study. Therefore, they have a high potential to be included in future analyses studying the effects of climate change and will help to improve predictions of vegetation changes.

## 1 INTRODUCTION

Changes in phenology are easily observable indicators for climate change, as especially spring phenology is susceptible to warming temperatures (Menzel & Fabian, 1999; Parmesan & Yohe, 2003; Root et al., 2003). Both experimental and observational studies showed changes in plant phenology with changing temperatures (Whittington, Tilman, Wragg, & Powers, 2015; Wolkovich et al., 2012), yet besides advances, also delays have been reported depending on the species and habitat observed (Bock et al., 2014; Fitter & Fitter, 2002; Menzel, 2000; Menzel et al., 2006; Root et al., 2003; Vitasse, Porté, Kremer, Michalet, & Delzon, 2009). Climate warming could thus enhance carbon uptake by lengthening canopy duration given sufficient precipitation (Liu et al., 2016; Menzel & Fabian, 1999) and lead to changes in the competitive balance by species‐specific differences in phenological sensitivity toward temperature (Vitasse, Porté, et al., 2009).

The onset of phenological phases mirrors the fundamental trade‐off between offspring development time and fitness of the parental plant (Bolmgren & Cowan, 2008). The optimal timing of bud burst is crucial for the survival of the plants: too early, the risk of frost damage is high, whereas too late, the potential growing season is not well used (Galvagno et al., 2013; Hänninen & Hari, 1996). This could also be true for flowering phenology, as reproductive shoots were found to be less frost resistant than vegetative tissue (Ladinig, Hacker, Neuner, & Wagner, 2013). Advances in flowering phenology might, however, lead to increased fecundity if there is no mismatch with pollinating insects (Baeten, Sercu, Bonte, Vanhellemont, & Verheyen, 2015; Sparks, Jeffree, & Jeffree, 2000). Overall, studies confirmed that the performance and abundance of species with an advanced phenology increased, whereas the performance of the others decreased (Baeten et al., 2015; Cleland et al., 2012; Hulme, 2011; Willis, Ruhfel, Primack, Miller‐Rushing, & Davis, 2008). Studies conducted along elevational gradients found significant changes in leaf phenology of tree species and species‐specific differences in temperature sensitivity (Schuster, Estrella, & Menzel, 2014; Schuster, Kirchner, Jakobi, & Menzel, 2014; Vitasse, Porté, et al., 2009). Herbaceous species are scarcely studied although they show strong changes in phenology (Bock et al., 2014; Fitter & Fitter, 2002; König et al., 2018; Menzel, 2000; Menzel et al., 2006; Root et al., 2003; Vitasse, Porté, et al., 2009; Vitasse et al., 2016).

Typically, studies report the onset of a phenological stage as day of the year, that is, first flowering day, FFD (e.g., Cornelius, Estrella, Franz, & Menzel, 2013; Cornelius, Leingärtner, et al., 2013; Fitter & Fitter, 2002). A complementary approach would be to measure the temperature accumulation reached to start flowering, or more specifically, the growing degree days to start FFD (GDD_FFD_; de Réaumur, 1735). We focussed on flowering phenology and assumed that there is not only a shift in FFD along the elevational gradient, but that populations from different elevations should also display different GDDs needed to reach FFD (GDD_FFD_) as besides temperature, photoperiod and adaptation to local climates were also found to influence phenology (e.g., Häkkinen, Linkosalo, & Hari, 1998; Heide, 1993; Migliavacca et al., 2008, 2011). Day length as a proxy for photoperiod differs during the year and peaks at midsummer. It is assumed to be constant for a given day of the year along the elevational gradients and thus its importance is negligible in our study. Its influence could however be tangible in a reduced time lag of FFD between higher and lower elevational sites compared to a purely temperature‐driven rate of change.

Although the response of flowering phenology to changing environmental conditions differs between species (Angert, Horst, Huxman, & Venable, 2010; Cleland, Chiariello, Loarie, Mooney, & Field, 2006; König et al., 2018; Morin, Roy, Sonie, & Chuine, 2010; Vitasse, Delzon, et al., 2009), growth forms (König et al., 2018), and cultivars (e.g., Bock et al., 2015), the reasons for these differing responses are not yet understood. Most studies have focussed on relating phenological shifts to temperature changes, but a few have explored the variation in species response with respect to their functional traits (e.g., Fitter & Fitter, 2002; König et al., 2018; Sun & Frelich, 2011). With our study, we want to shed light on the association between flowering phenology and plant functional traits. We focus on two different aspects. First, we analyze whether FFD is related to functional traits. In a second approach, we investigate whether the species‐specific differences in the intensity of observed shifts in FFD along elevational gradient can be explained with functional traits. We assume that a species' trait might enable it to adapt to changing environments as traits might be linked to either earlier or later flowering. Thus, we assume several traits to be related to FFD, that is traits related to (1) plant performance (specific leaf area, SLA), (2) leaf biochemistry (leaf C, N, P, K, and Mg content), and (3) water‐use efficiency (stomatal pore area index [SPI] and stable carbon isotopes concentration). Specific leaf area is widely accepted as a good approximation of plant growth rate (Garnier, 1992; Pérez‐Harguindeguy et al., 2013) and indicates competitive ability and environmental tolerance (Poorter, Niinemets, Poorter, Wright, & Villar, 2009; Reich, Walters, & Ellsworth, 1997). It was found to have a negative relation with flowering time, that is, early flowering plants had higher growth rates (Sun & Frelich, 2011), and higher SLA was found to be associated with stronger phenological shifts in herbaceous plants (König et al., 2018). Area‐based leaf carbon content (C_area_) is a measure for the plants investment in structural components and photosynthetic sugar accumulation, thus early flowering plants might contain less C_area_ as it accumulates over time (Larcher, 1994), whereas shifts in phenology should be less pronounced due to a trade‐off between the investment in growth and reproduction (Bolmgren & Cowan, 2008). Biochemical traits related to enzyme content and thus to photosynthesis rates (N_area_ and P_area_, Mg_area_; Bond, Farnsworth, Coulombe, & Winner, 1999; Bucher, Bernhardt‐Römermann, & Römermann, 2018; Evans, 1989; Feng & Dietze, 2013; Larcher, 1994) should be higher in early flowering plants, as they are important to start metabolism rapidly. Leaf nutrients should also influence the strength of the shift in FFD as plant performance is positively associated with phenological shifts (Cleland et al., 2012; Willis et al., 2008). Potassium (K) is an important ion for the activation of enzymes (e.g., in photosynthesis and nitrate reductase) and for electrochemical interactions such as the transmembrane potential difference, osmotic regulation, and stomatal movements and was shown to have a big influence on leaf unfolding (Jochner et al., 2013). Accordingly, we also expect early flowering to be correlated with higher K values and a positive influence in the strength of shifts in FFD. Stomata are the mediators of gas exchange as they govern the uptake of CO_2_ and the regulation of transpiration (Chaerle, Saibo, & Van Der Straeten, 2005; Roelfsema & Hedrich, 2005) and are associated with actual conductance and maximum photosynthesis rates (Bucher et al., 2016; Sack, Cowan, Jaikumar, & Holbrook, 2003). We expect a negative relationship of stomatal traits and FFD as they relate to a more conservative strategy and leaves might not be fully developed but higher SPI should strengthen the shift in FFD via its association with performance. The carbon isotope discrimination (Δ^13^C) can be used as a proxy for water‐use efficiency and the relative internal CO_2_ concentrations and relates to photosynthesis rates (Bucher et al., 2018; Farquhar, Ehleringer, & Hubick, 1989; Pérez‐Harguindeguy et al., 2013). As with early flowering, plants might not be fully developed and water conservation is not as important as in summer, we expect lower water‐use efficiency in these individuals yet a positive influence on shifts in FFD. Table 1 gives an overview on hypothesized relationships between plant traits and flowering phenology.

**Table 1:** Overview of leaf functional traits measured and analyzed in our study as well as their ecological significance and their hypothesized relationship with first flowering day (FFD) and shifts in FFD

Trait	Abbreviation	Unit	Ecological significance	Link to flowering phenology
Area‐based leaf carbon content	C_area_	g/m^2^	Structural compounds, photosynthesis product (Larcher, 1994)	Associated with later flowering as C_area_ accumulates over time, shifts should be less pronounced because of trade‐off between growth and reproduction
Carbon isotope discrimination	Δ^13^C	‰	Water‐use efficiency, internal CO_2_ concentration (Bucher et al., 2018; Farquhar et al., 1989; Pérez‐Harguindeguy et al., 2013)	Associated with later flowering as water‐use efficiency is less important early in the year, shifts less pronounced due to conservative strategy of plants
Area‐based leaf nitrogen content	N_area_	g/m^2^	Proxy for photosynthetic capacity/ RubisCO content (Bond et al., 1999; Bucher et al., 2018; Evans, 1989; Larcher, 1994)	Associated with earlier flowering and stronger shifts as flowering is associated with high metabolic activity
Area‐based leaf phosphorus content	P_area_	g/m^2^	Important in metabolism and synthesis (Feng & Dietze, 2013; Larcher, 1994)	Associated with earlier flowering and stronger shifts as flowering is associated with high metabolic activity
Area‐based leaf potassium content	K_area_	g/m^2^	Activation of Enzymes, electrochemistry, osmotic potential (Larcher, 1994)	Associated with earlier flowering and stronger shifts in flowering phenology (Jochner et al., 2013)
Area‐based leaf magnesium content	Mg_area_	g/m^2^	Important in metabolism, osmotic potential, photosynthesis (Larcher, 1994)	Associated with earlier flowering but weaker shifts because of trade‐off between growth and reproduction
Stomatal pore area index	SPI	–	Potential conductance, photosynthesis rates (Bucher et al., 2016; Sack et al., 2003)	Associated with later flowering and shifts less pronounced because of trade‐off between growth and reproduction
Specific leaf area	SLA	m^2^/kg	Proxy for growth rate (Garnier, 1992; Pérez‐Harguindeguy et al., 2013)	Associated with earlier flowering (Sun & Frelich, 2011) and stronger shifts in flowering phenology (König et al., 2018)

We used elevational gradients to investigate the association between changes in flowering phenology in response to changing environments (i.e., air temperature) and plant functional traits. More specifically, we monitored flowering phenology and measured plant functional traits concurrently of ten herbaceous species in the Bavarian Alps. To account for spatiotemporal variations, we monitored these species along two elevational gradients during two consecutive growing seasons. We addressed the following questions:

1. Do herbaceous plants react analogously to tree species and delay the onset of FFD along the elevational gradient and are there differences among species, between gradients and between years?
2. Does flowering time have an influence on the intensity of shifts in FFD, that is, do early flowering species shift their phenology stronger than late‐flowering species?
3. Does GDD_FFD_ as an ecological measure of phenological changes also change along the elevational gradient?
4. Can plant functional traits be used to capture and thus explain differences in FFD as well as the intensity with which species change their FFD along the elevational gradients?

Studying the phenology of herbaceous plants complements our knowledge on vegetation responses to environmental changes, and the output of this study may serve as a first step to understand the role of trait values for variations in flowering phenology and thus to improve predictions of vegetation changes.

## 2 MATERIAL AND METHODS

### 2.1 Study area and selected species

The study area was located in the montane to subalpine belt of the northern limestone Alps in the area of Garmisch‐Partenkirchen. Two south‐facing elevational gradients (along “Kramer” and “Kreuzeck” mountain; see Schuster, Estrella, et al., 2014) were set up ranging from 700 to 1,800 m a.s.l. and 800—1,700 m a.s.l., respectively. Mean annual temperatures decrease with a lapse rate of −0.55°C/100 m (Kirchner et al., 2013). According to their occurrence along a wide elevational range (as queried in the regional vegetation database of Ewald (2012) and based on personal observations), ten perennial hemicryptophytes were chosen for this study. We selected *Aposeris foetida* (L.) Less., *Aster bellidiastrum* (L.) Scop., *Buphthalmum salicifolium* L., *Carduus defloratus* L., *Knautia dipsacifolia* Kreutzer, *Lotus corniculatus* L., *Mercurialis perennis* L., *Phyteuma orbiculare* L.*, Trifolium pratense* L., and *Potentilla erecta* (L.) Raeusch. Habitat conditions were kept as constant as possible along the elevational gradient following the species' optimum conditions, for example, shade species such as *M. perennis* were always collected from the forest edge whereas sun‐loving species such as *B. salicifolium* were collected from open habitats. The Table S1 in Supporting Information gives an overview on mean FFD and GDD_FFD_ of all species.

### 2.2 First flowering day

Phenology was monitored in two consecutive years (2012 and 2013). The first day when a species was found to display fully developed flowers (FFD) was recorded following Fitter and Fitter (2002). Populations of the selected species were monitored weekly along the two gradients every 100‐m increase in elevation during the growing season (beginning of April until beginning of November). We monitored up to three replicate populations per elevational band, which we then averaged for consecutive analyses and calculated a mean value for each species per elevational band, year, and gradient.

### 2.3 Temperature records and growing degree days of FFD (GDD_FFD_)

Temperature was recorded at 10‐min intervals with automatic weather stations (HOBO V2 with radiation shield, Onset, Bourne, MA, USA) installed at all sites along the elevational gradients (22 in total). The GDD were determined for the FFD of each species and site (referred to as GDD_FFD_) following equation 1 starting from the 1st of January.

(1) $${\text{GDD}}_{\mathrm{FFD}}\;=\;\sum \frac{T_{\max}\;+\;T_{\min}}{2}-T_{\mathrm{base}}$$

*T*_max_ is the daily maximum temperature, *T*_min_ the daily minimum temperature, and *T*_base_ the base temperature above which plant growth could occur, thus all negative values were deleted before summing up. In our study, we used a base temperature of 5°C. We checked that (*T*_max_ + *T*_min_)/2 was highly correlated with *T*_mean_ (*r* = .997, *p *<* *.001).

### 2.4 Plant functional traits

For each species and in both years, all populations were characterized with respect to traits associated with (1) plant performance (SLA), (2) biochemical traits (leaf C, N, P, K, and Mg content), and (3) water‐use efficiency (SPI and Δ^13^C). All traits were measured on five fully flowering individuals per elevational band and gradient following standardized methods (Pérez‐Harguindeguy et al., 2013) to characterize the populations' trait values. Table 1 gives an overview on traits and their ecological functions.

Specific leaf area is defined as the ratio of fresh leaf area to dry mass (m^2^/kg). For each focal individual, two replicate leaves were collected. Dry mass was recorded separately using a fine scale. Leaf area was quantified by scanning fresh leaves (CanoScan LiDE110, Cannon, Tokyo, Japan). From the scans, leaf area was retrieved and SLA was calculated in R (R Core Team 2016) using the R‐Package LeafTraits (M. Bernhardt‐Römermann, unpublished).

For the analysis of chemical compounds, the leaves were pooled per population. Mass‐based leaf carbon concentration (C_area_), carbon isotope composition, and mass‐based leaf nitrogen concentration (N_area_) were measured using 0.2 mg of dried, milled leaf tissue weighed into tin capsules, and combusted in an elemental analyzer (NA 1110, Carlo Erba, Milan, Italy) coupled to an isotope ratio mass spectrometer via a Conflow interface. The relative carbon isotope ratio (δ^13^C) was calculated and the discrimination (Δ^13^C) was calculated according to Farquhar et al. (1989). We used δ^13^C in leaves and δ^13^C in the atmosphere 1 month before sampling of the leaf to calculate Δ^13^C as described in Bucher et al. (2016). Leaf nitrogen concentration and leaf carbon concentration per unit leaf area (μg/mm^2^) were calculated by dividing the mass‐based values of the respective compounds by SLA. Similarly, we determined leaf P_area_, K_area,_ and Mg_area_ content via an inductively coupled plasma mass spectrometry (iCAP Qc ICP‐MS; Thermo Fisher Scientific GmbH, Bremen, Germany) following DIN EN ISO 17294‐2 after a high pressure (30 bar), acid digestion of the samples at high temperatures (180°C).

Stomatal traits were investigated using the clear nail polish method as described in Hilu and Randall (1984). Two imprints from the abaxial and one from the adaxial side of one leaf of each focal individual were taken to assess stomatal density on two fields of view and stomata size on two replicate stomata per field of view. Using these measures, the dimensionless SPI was calculated as proposed by Sack et al. (2003) as:

(2) $$\text{SPI}\;=\;{\left(\text{guard cell length}\right)}^{2}\text{stomatal density}$$

### 2.5 Data analyses

To characterize spatiotemporal variations in abiotic conditions, we compared the mean temperatures of all temperature logger sites between (1) the two gradients, (2) the 2 years separately, and (3) between the months of each year. We used the Welch two‐sample *t* test or the Wilcoxon rank sum test with continuity correction after having checked for normal distribution and homogeneity of variance.

To investigate whether herbaceous plants shifted their FFD along the gradient, we analyzed whether FFD differed along the elevational gradient between species, gradients (along Kramer or Kreuzeck), year of observation, or a combination of these variables. We set up linear models using FFD as dependent variable and species, gradient, elevation, and year, as well as all twofold interactions thereof as explanatory variables. Full models were subsequently simplified using stepwise backwards selection until the least significant adequate model was found (Crawley, 2012). In all cases, variances were homogeneous and the residuals normally distributed.

To test whether flowering time (early or late flowering) had an influence on the shifts of phenology along the gradient, we set up a linear model with the rate of change along the elevational gradient as calculated for each species, year, and gradient as dependent variable and FFD as explanatory variable.

To test whether we find similar patterns for GDD_FFD_, the same models analyzing the difference GDD_FFD_ in along the elevational gradient between species, gradients, and year of observation were set up using GDD_FFD_ as dependent variable instead of FFD.

To assess whether FFD is associated to plant traits, we set up two different models including traits besides elevation, gradient, and year. One model focusses on absolute values in FFD, the other focusses on species‐specific shifts in FFD along the elevational gradient. In the first model approach, we ignored species identity because we were merely interested in the functional association between trait values and FFD. Preliminary analyses (not shown) revealed that each species, included as a covariate, showed clear species‐specific patterns thus including species identity as a covariate would have covered the relative importance of the trait values to explain FFD. This approach was assessed to be sound as for all FFD data points, in situ measurements of traits on all populations were available. In the first FFD‐trait model, we included FFD as dependent variable and trait values, elevation, and gradient as explanatory variable. In the second model, we used the species‐specific slope of the relationship between FFD and elevation (intensity of shifts, i.e., based on estimates from the regression of FFD along the elevational gradient) as dependent variable and the mean trait values for each species, year, and gradient as explanatory variables. In both models, we used boosted regression trees (BRT) as described in Elith, Leathwick, and Hastie (2008) because they provide insights into the relationships between predictor and dependent variables. The BRT output provides importance measures for each predictor variable included (i.e., the sensitivity of a trained model to each of the predictors) and partial dependency plots that marginalize (integrate) over the effect of all other predictor variables (Friedman, 2001). Thus, the relationship of a predictor—as independent of all other predictors included—can be displayed. Boosted regression trees is a flexible regression technique based on machine learning. BRTs do not require a priori information on functional relationships, but “learn” the relationships between the response and its predictors by identifying patterns in the data. Gaussian error distribution was used as well as a bag fraction (the fraction of the training set observations randomly selected to propose the next tree in the expansion) of 0.5, tree complexity of 2, and a learning rate of 0.001. We simplified the models and used the cross‐validation error (cv) as predictor of the goodness of fit. For all variables, if included in the simplified models, the relative importance (%) is given.

For calculation and simplification, the “gbm” package was used (Ridgeway, 2015). All analyses were conducted in R 3.3.0 (R Core Team, 2016).

## 3 RESULTS

### 3.1 Abiotic conditions and flowering phenology

Temperatures were not significantly different between gradients (*W* = 30,821, *p* = .18) or between 2012 and 2013 (*W* = 43,062, *p* = .43), yet monthly temperatures differed. In February, mean daily temperatures were significantly warmer in 2013 than in 2012 (*t* = −4.89, *p *<* *.001). March, May, and June temperatures were significantly colder in 2013 than in 2012 (March: *t* = 13.49, *p *<* *.001; May: *t* = 5.87, *p *<* *.001; June: *t* = 4.24, *p *<* *.001). July (*t* = −5.59, *p *<* *.001) and October (*t* = −3.82, *p *<* *.001) were warmer in 2013 than in 2012. August (*t* = 2.44, *p *<* *.05) was a little colder in 2013 than in 2012 and November was significantly colder in 2013 (*t* = 11.87, *p *<* *.001) whereas December was significantly warmer in 2013 than in 2012 (*t* = −9.09, *p *<* *.001). For a graphical display, please see Figure S1.

**Figure 1:**
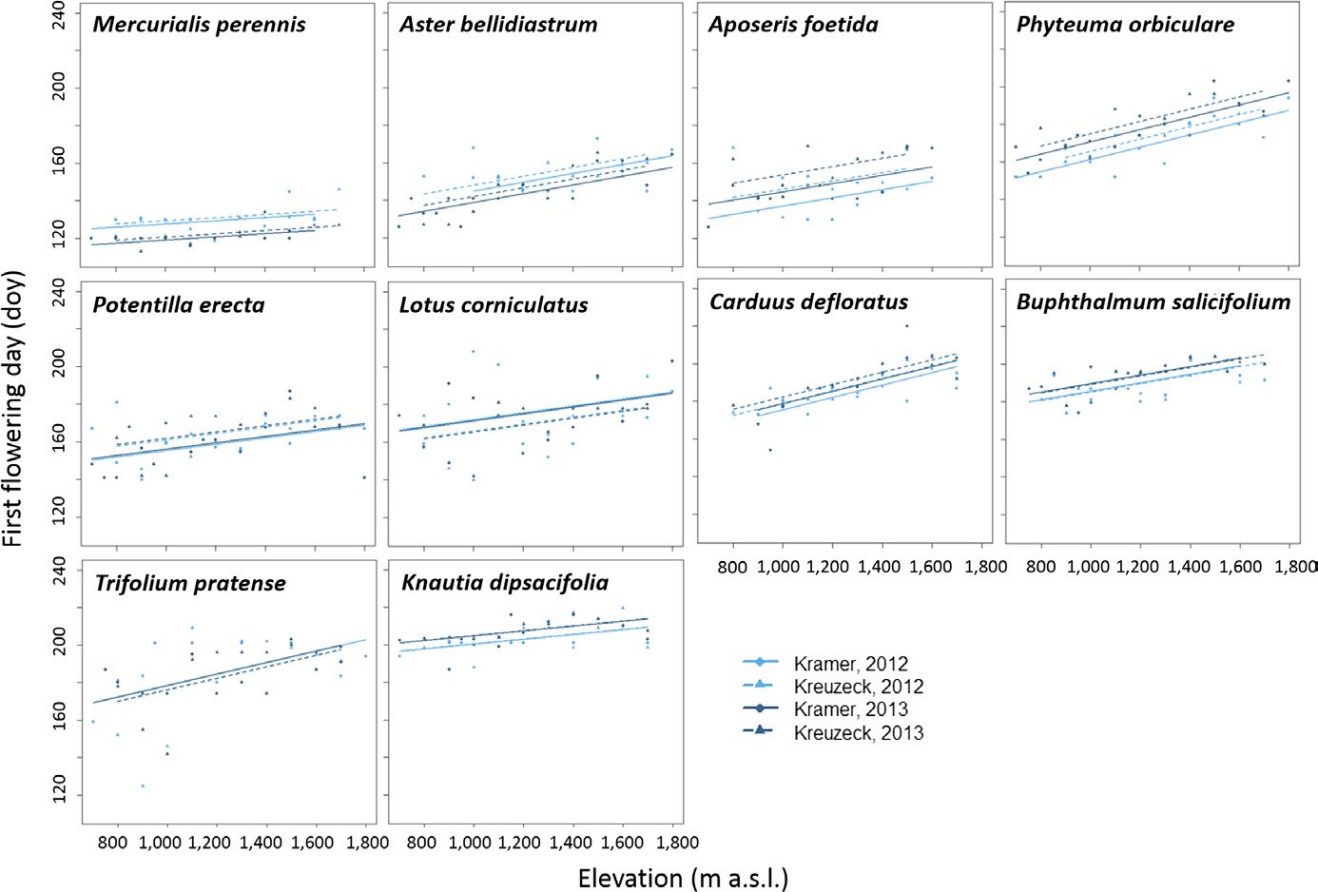
First flowering day given in day of the year (doy) along two elevational gradients (Kramer vs. Kreuzeck) as depending on species and year (2012 vs. 2013) as well as the interactions of elevation:species, species:gradient, and species:year. Light blue indicates 2012, dark blue indicates 2013. Solid lines and circles represent Kramer, dashed lines, and triangles represent Kreuzeck gradient

The species which flowered earliest were *M. perennis* (mean FFD = 126.1, beginning of May) and *A. foetida* (mean FFD = 149.4, end of May) whereas *B. salicifolium* (mean FFD = 192.2, mid‐July) and *K. dipsacifolia* (mean FFD = 205.4, end of July) flowered latest. All species showed a delay in FFD along the elevational gradient in a species‐specific way (species effect *p *<* *.001; Figure 1). The flowering phenology differed between Kreuzeck and Kramer (*p *<* *.001) and between years (*p *<* *.001; *R* ^2^ = .85, *F*_39,371_ = 52.3, *p *<* *.001). *M. perennis* showed the weakest change with only 0.84 day 100/m while *C. defloratus* showed the strongest change with 3.28 day 100/m. Apart from *L. corniculatus*,* B. salicifolium,* and *K. dipsacifolia,* all species flowered later on Kreuzeck than on Kramer (for details, see Table S1), thus flowering per se was marginally later on Kreuzeck than on Kramer (*W* = 18,909, *p *<* *.1). The intensity of shifts in FFD along the elevational gradient (slope) was positively related to FFD (*R* ^2^ = .14, *F*_1,38_ = 6, *p *<* *.05), indicating that late‐flowering species were more responsive in their phenology than early flowering species.

**Figure 2:**
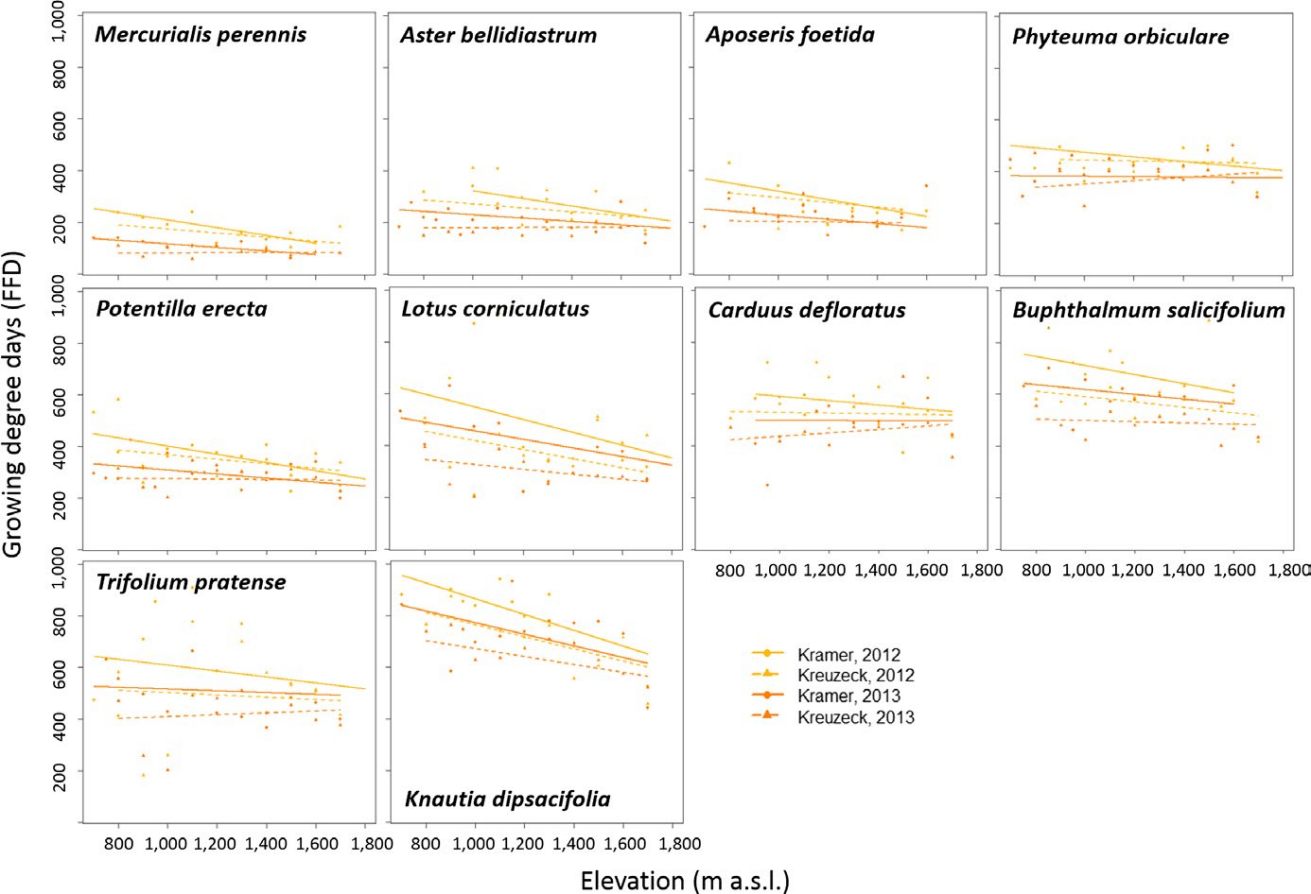
Growing degree days of first flowering day along two elevational gradients (Kramer vs. Kreuzeck) as depending on species and year (2012 vs. 2013) as well as the interactions of elevation:species, species:gradient, and species:year. Yellow indicates 2012, orange 2013. Solid lines and circles represent Kramer, dashed lines, and triangles represent Kreuzeck

Species also differed in their FFD response to temperature accumulation as expressed by GDD_FFD_ (Figure 2). GDD_FFD_ depended on elevation, species, gradient, and year and the interaction of species with elevation, gradient, and year (*R* ^2^ = .82, *F*_32,369_ = 54.1, *p *<* *.001). All species despite *C. defloratus* in 2013 showed a decrease in GDD_FFD_ with increasing elevation.

### 3.2 Relation of FFD to plant functional traits

**Figure 3:**
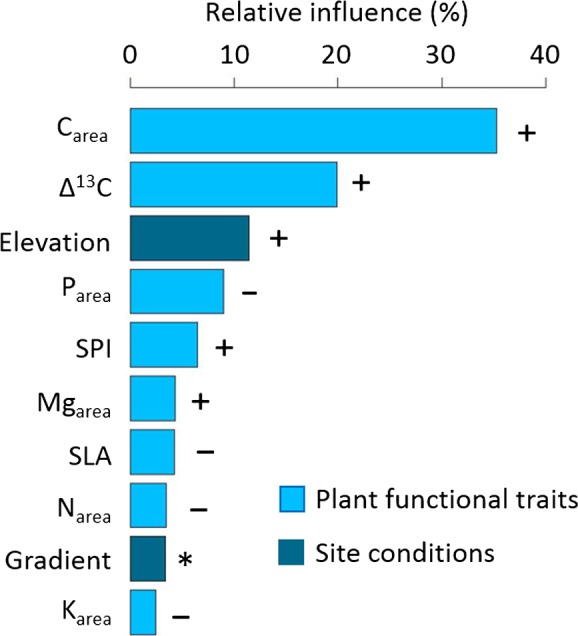
Relative importance of plant functional traits and elevation and gradient as integrating factors over site conditions on first flowering day (FFD) as deduced from boosted regression trees. C_area_: Carbon per unit leaf area (g/m^2^), Δ^13^C: Discrimination of ^13^C (‰), elevation (m a.s.l.), P_area_: Phosphorus per unit leaf area (g/m^2^), SPI: Stomatal pore area index (×10^2^), Mg_area_: Magnesium per unit leaf area (g/m^2^), SLA: Specific leaf area (m^2^/kg), N_area_: Nitrogen per unit leaf area (g/m^2^), gradient: Kramer and Kreuzeck, and K_area_: Potassium per unit leaf area (g/m^2^). For the partial dependency plots displaying the relationships of the explanatory variables to FFD in detail, please see Appendix S2

The results of the BRTs (cv = 0.75) showed that plant functional traits had a higher relative importance for explaining FFD (in total 85.1%) than abiotic conditions as represented by elevation, gradients, and years (in total 14.8%). Elevation, accounting for 11.5% of the variation, was only ranked third among the explanatory variables, gradient was of minor importance (3.3%) and year had no influence on FFD in the final model. The variables being most closely related to FFD were C_area_ (35.3%) and Δ^13^C (19.9%; see Figure 3), both showing a positive relationship with FFD, that is., with increasing trait values, FFD was delayed. SPI (6.5%) and Mg_area_ (4.3%) were also positively related to FFD. In contrast, P_area_ (9%), SLA (4.2%), N_area_ (3.5%), and K_area_ (2.4%) were negatively related to FFD. For detailed information on how traits influence FFD along the elevational gradient, partial dependency plots are presented in Figure S2. The regression of predicted versus observed values suggests a good model fit and can be found in Figure S3.

**Figure 4:**
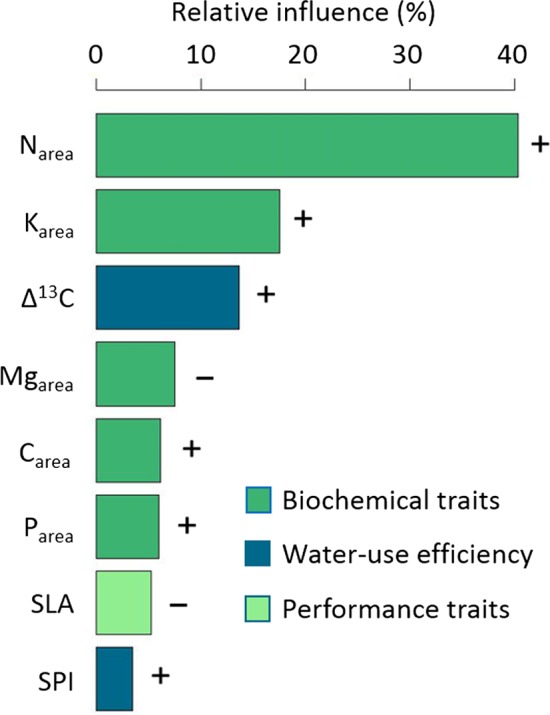
Relative importance of plant functional traits on the shifts of first flowering day (FFD) along the elevational gradient as deduced from boosted regression trees. N_area_: Nitrogen per unit leaf area (g/m^2^), K_area_: Potassium per unit leaf area (g/m^2^), Δ^13^C: Discrimination of ^13^C (‰), Mg_area_: Magnesium per unit leaf area (g/m^2^), C_area_: Carbon per unit leaf area (g/m^2^), P_area_: Phosphorus per unit leaf area (g/m^2^), SLA: Specific leaf area (m^2^/kg), and SPI: Stomatal pore area index (×10^2^). For the partial dependency plots displaying the relationships of the explanatory variables to the shifts in FFD in detail, please see Appendix S4

The second BRT model showed that especially high N_area_ (40.3%), K_area_ (17.5%), and Δ^13^C (13.7%; see Figure 4) were associated with stronger shifts in phenology along the elevational gradient (cv = 0.52). In addition to that, higher C_area_ (6.2%), P_area_ (6.0%), and SPI (3.5%) values lead to stronger shifts in FFD whereas higher Mg_area_ (7.5%) and SLA (5.3%) led to less intense changes of phenology. For detailed information on how traits influence the shifts of FFD along the elevational gradient, partial dependency plots are presented in Figure S4. The regression of predicted versus observed values suggests a good model fit and can be found in Figure S5.

## 4 DISCUSSION

Our results clearly confirm that also in herbaceous species, flowering phenology was delayed with increasing elevation and thus with decreasing mean annual temperatures in a species‐specific intensity, which confirms studies that analyzed these relations in woody plants (Schuster, Estrella, et al., 2014; Schuster, Kirchner, et al., 2014; Vitasse, Porté, et al., 2009). We found that GDD_FFD_ decreased with elevation, which indicates both, a dependence on temperatures but also on photoperiod. A very striking result is that traits were more important to explain changes in FFD compared to elevation, especially traits related to water‐use efficiency and growth of the leaves had the highest relative importance to explain variations in flowering phenology. Changes in FFD along the elevational gradient were also associated with plant traits and especially higher N_area_, K_area,_ and Δ^13^C values led to stronger shifts of phenology along elevational gradients.

We could not confirm previous studies which found that advances of flowering phenology are stronger the earlier plants flowered (Fitter & Fitter, 2002; Menzel et al., 2006; Miller‐Rushing & Primack, 2008), but the opposite was true for our dataset, however, revealed by the space for time approach. We also observed differences in FFD between the two gradients and years, which are likely due to differing abiotic conditions or local adaptations of the plant populations, as the years differed in mean monthly temperatures but the gradients did not. Similar results could be observed by Ziello, Estrella, Kostova, Koch, and Menzel (2009) on a larger scale, who found not only a species‐specific delay of phenology with increasing elevation but also detected regional differences, namely a stronger response in the northern than in the southern Alps. Also, Cornelius, Estrella, et al. (2013) detected differences in phenological responses between observational sites in the Alps. Hopkins law states that species should delay their phenology by 3.3 day 100/m (Fitzjarrald, Acevedo, & Moore, 2001; Vitasse, Delzon, et al., 2009) yet only three species reacted almost as strongly as predicted. *C. defloratus* shifted its phenology by 3.28 day 100/m, *P. orbiculare* by 3.26 day 100/m, and *T. pratense* by 3.06 day 100/m, the other species were less responsive. Other observational studies in alpine grasslands showed an average delay of 3.8 day 100/m (Cornelius, Estrella, et al., 2013; Cornelius, Leingärtner, et al., 2013). Interestingly, for *A. foetida* and *M. perennis,* the reaction was much less pronounced compared to the observations on these species reported by Cornelius, Estrella, et al., 2013 and Cornelius, Leingärtner, et al., 2013; 2.19 day 100/m instead of 3.7 day 100/m for *A. foetida* and 0.85 day 100/m compared to 3.5 day 100/m for *M. perennis*, respectively). This illustrates highly variable regional patterns. However, this reaction seems to be quite conserved within the small‐scale region as the slope of FFD along the elevational gradients did not vary between years and the two gradients of our study.

Early plants flowered later in 2012 than in 2013 whereas this trend was reversed with later flowering species. This might be due to much colder February temperatures in 2012, which inhibited plant growth early in the season, whereas spring to early summer (mainly March, May, and June) was warmer in 2012. In previous studies, early flowering subarctic species showed less intraspecific variability in FFD on an elevational gradient via a tighter coupling of phenology and climatic cues and were more variable when comparing several years due to interannual changes in climate (Lessard‐Therrien, Bolmgren, & Davies, 2014). Again, for herbaceous species, we could not confirm this trend with our analysis as variability of the 2 years and two gradients did not change with elevation (Figure 1).

The detected negative relationship between GDD_FFD_ and elevation might indicate a slight influence by photoperiod as assessed via day of the year, which was assumed to be constant throughout the elevational gradient. It might advance the phenology of higher elevational species as compared to lower elevational species leading to this negative relationship. This confirms the finding of Heide (1993), who stated that longer days decreased the thermal time to budburst. Experimental studies of 33 plant species in the Central Alps demonstrated that around 50% of the species might be under strong limitations by photoperiod in their spring phenology (Keller & Körner, 2003) which is pronounced in the case of very early snowmelt (Galvagno et al., 2013; Migliavacca et al., 2011). Differently, Vitasse et al. (2016) found that alpine phenology was determined mostly by snowmelt date and they found no major limitation by photoperiod as assessed via day of the year. Unfortunately, we do not have reliable snowmelt data for our dataset. Previous studies reported a negative relationship between GDD requirements and chilling accumulation and a decline of GDD with latitude (Fu et al., 2014; Laube et al., 2014; Pellerin, Delestrade, Mathieu, Rigault, & Yoccoz, 2012). However, this might not only be due to unfulfilled chilling requirements (e.g., Laube et al., 2014) but also to a lower *T*_base_ of species which might decrease along the elevational gradient. The fact that GDD_FFD_ is declining with elevation is contrasting the results for budburst in woody species, namely *Fraxinus excelsior* L., *Betula pendula* Roth, and *Larix decidua* Mill. and leafing for *B. pendula* and *F. excelsior* (Pellerin et al., 2012) but is in line with results shown for other temperate trees in Hunter and Lechowicz (1992). Moreover, FFD is both showing a delay with increasing elevation and a decline of GDD_FFD_, suggesting that our species are controlled by multiple factors at the same time. Figure 2 suggests that the variability in GDD_FFD_ decreased with increasing elevation with the same slope for all species, which could also be confirmed when testing this relation in a linear model (*R* ^2^ = .23, *F*_10,99_ = 2.95, *p *<* *.01). This result might indicate a stronger abiotic filtering at higher elevations, that is. stronger selection for individuals, which respond to a temperature cue than in lower elevational sites.

In addition, we found that functional traits were strongly associated with the FFD and were even more important to explain variations than elevation. Most influential was C_area_ as a measure of plants' investment in structural components and photosynthetic sugar accumulation (Larcher, 1994). It was associated with later dates of FFD, which indicates a trade‐off between offspring development time and fitness of the parental plants (Bolmgren & Cowan, 2008). Carbon isotope discrimination as a time‐integrated estimate of internal CO_2_ concentration and intrinsic water‐use efficiency (Farquhar et al., 1989; Pérez‐Harguindeguy et al., 2013) was also very important to explain variations in FFD. Higher discrimination and thus lower water‐use efficiency were associated with later dates of first flowering. Δ^13^C is increased when either stomata are open or photosynthesis rates are low (Bucher et al., 2018; Farquhar et al., 1989). Our results indicate that higher photosynthesis rates are associated with earlier FFD, which is supported by the fact that nitrogen and phosphorus, both linked to enzyme content and metabolism were also associated with earlier FFD. Phosphorus is essential for plant growth but is often limited under natural conditions (Vitousek, Porder, Houlton, & Chadwick, 2010), which has been documented for *Picea abies* (L.) Karst and *Fagus sylvatica* L. in the same research area (Ewald, 2000; Mellert & Ewald, 2014). In horticulture, phosphorus is often used to induce flowering. Previous studies found a link between soil phosphorus content and phenology in *F. sylvatica* where high soil phosphorus led to higher tissue P content and advanced leaf unfolding but not bud break (Yang, Zavišić, Pena, & Polle, 2016), yet there was no significant relationship of soil P and leaf P_area_ in our study (analyses not shown). Higher SPI was associated with later flowering. Römermann, Bucher, Hahn, and Bernhardt‐Römermann (2016) found highest SPI during midseason within a species, and Bucher et al. (2016) displayed the positive relationship between SPI and photosynthesis rates. Contrasting to our hypothesis, Mg_area_, which is essential for photosynthesis was related to a later onset of phenology, indicating higher maturity of the leaves. As opposing other leaf biochemical compounds, Mg is mostly located in chlorophyll and rarely found in other enzymes which explains the deviation from the results for N_area_ and P_area_, the latter two being related to earlier FFD. High SLA is linked to high growth rates (Pérez‐Harguindeguy et al., 2013) and had an advancing effect on FFD. The relationship of SLA to later FFD confirms studies by Sun and Frelich (2011) which might indicate the trade‐off between flowering phenology and offspring development time and fitness of the parental plant (Bolmgren & Cowan, 2008). Potassium is involved in cell extension, membrane function, and stability and leads to earlier onset of FFD in our study, confirming studies demonstrating a strong correlation of spring phenology of *Betula pubescens* Ehrh. with potassium content (Jochner et al., 2013).

Moreover, we could demonstrate that traits are associated with shifts in phenology along the elevational gradient and are thus influencing the ability of species to adapt to climate change. We found that especially higher nitrogen and potassium content were linked to stronger phenological shifts as well as higher Δ^13^C and thus lower water‐use efficiency. Nitrogen, being related to enzyme content and photosynthesis rates (Bucher et al., 2018; Evans, 1989), had highest importance for the ability to shift FFD which strengthens the claim of shifts in FFD being directly related to plant performance (Cleland et al., 2012; Willis et al., 2008). In our study, we found that SLA was related to less pronounced shifts in FFD which is opposing findings by König et al. (2018) for herbaceous plants on a global scale. However, SLA had a low relative importance in our model which emphasizes that different mechanisms could act on different scales. As SLA is also driven by leaf thickness, the investment in thicker leaves and thus higher resistance and lower growth rates seem to slow down phenological shifts. We also hypothesized weaker shifts with increasing C_area_ and stronger shifts with increasing Mg_area_ which could not be found in our data. This might be due to the fact that carbon is not only located in structural compounds, an aspect which is also captured indirectly via SLA but also accumulates during photosynthesis where CO_2_ is fixed via RubisCO. Thus, it captures plant performance as well, which is in line with our findings for N_area_, P_area,_ K_area_, and Mg_area_. On the other hand, it reduces the strength of shift in FFD similar to SLA and indicates the investment in long‐lasting leaf organs and thus a more conservative life strategy. This indicates that plants displaying higher photosynthesis and metabolism rates are able to shift their phenology stronger than plants with lower performance. Traits may thus be used in vegetation models as proxy to describe the ability of species to respond to changing climate.

Our findings indicate a relationship between FFD and traits, yet further research is needed concerning the intraannual variability and its association with phenology, as proposed by Römermann et al. (2016). We could demonstrate that traits related to water‐use efficiency and growth of the leaves had the highest relative importance to explain differences in flowering phenology surpassing even the explanatory power of temperature on the variation in FFD and influenced the ability to shift phenology. This study represents a first indication to analyze species‐specific changes to changing environmental conditions and will help to better grasp the effects of changing temperatures on vegetation changes and improve future predictions.

## CONFLICT OF INTEREST

None declared.

## AUTHOR'S CONTRIBUTION

SFB and CR designed the study and the methodology. SFB collected the data; AM provided the temperature data; SFB, CR, AM, JE, PK, and MM discussed and analyzed the data. All authors contributed valuably to the drafts and gave final approval for publication.
