# Supplementary material for: Traits and climate are associated with first flowering day in herbaceous species along elevational gradients
Source: Ecol Evol. 2017 Dec 20;8(2):1147–58. doi: 10.1002/ece3.3720 (PMC5773311; doi:10.1002/ece3.3720)
Supplement: Supplementary file 1 [file ECE3-8-1147-s001.docx]

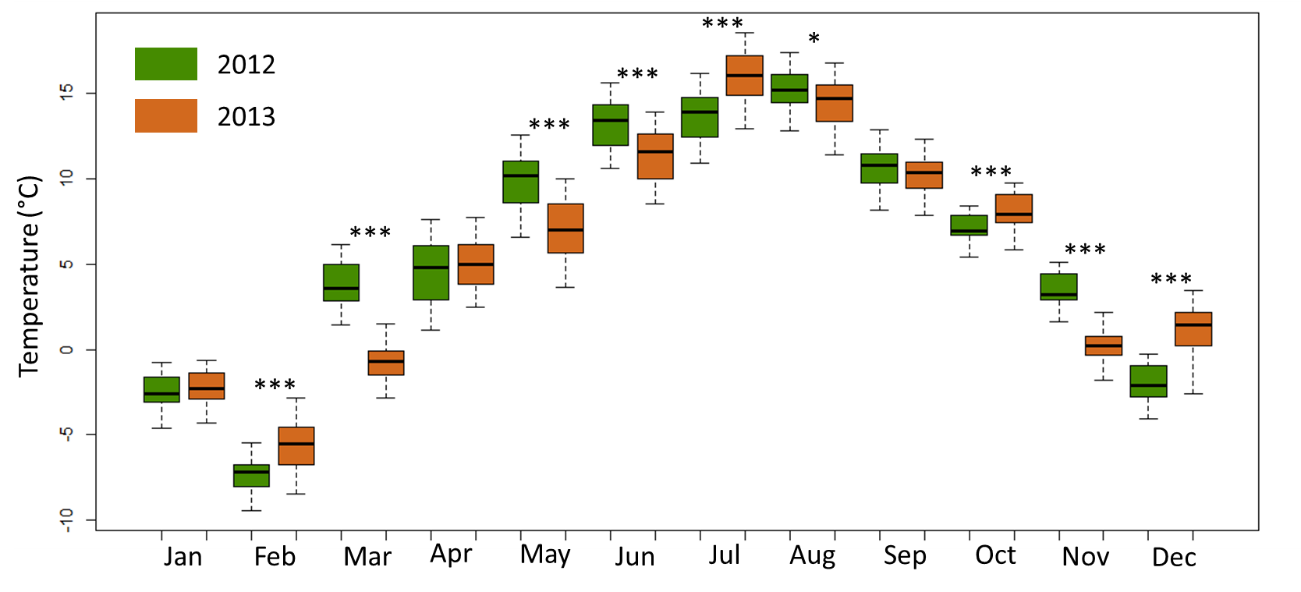


Figure S1: Mean monthly temperature of all weather stations in 2012 (displayed in green) and 2013 (displayed in brown). Asterisks indicate significant differences between the two years: `***´: p < 0.001, `*´: p < 0.05.
