# Supplementary material for: Traits and climate are associated with first flowering day in herbaceous species along elevational gradients
Source: Ecol Evol. 2017 Dec 20;8(2):1147–58. doi: 10.1002/ece3.3720 (PMC5773311; doi:10.1002/ece3.3720)
Supplement: Supplementary file 4 [file ECE3-8-1147-s004.docx]

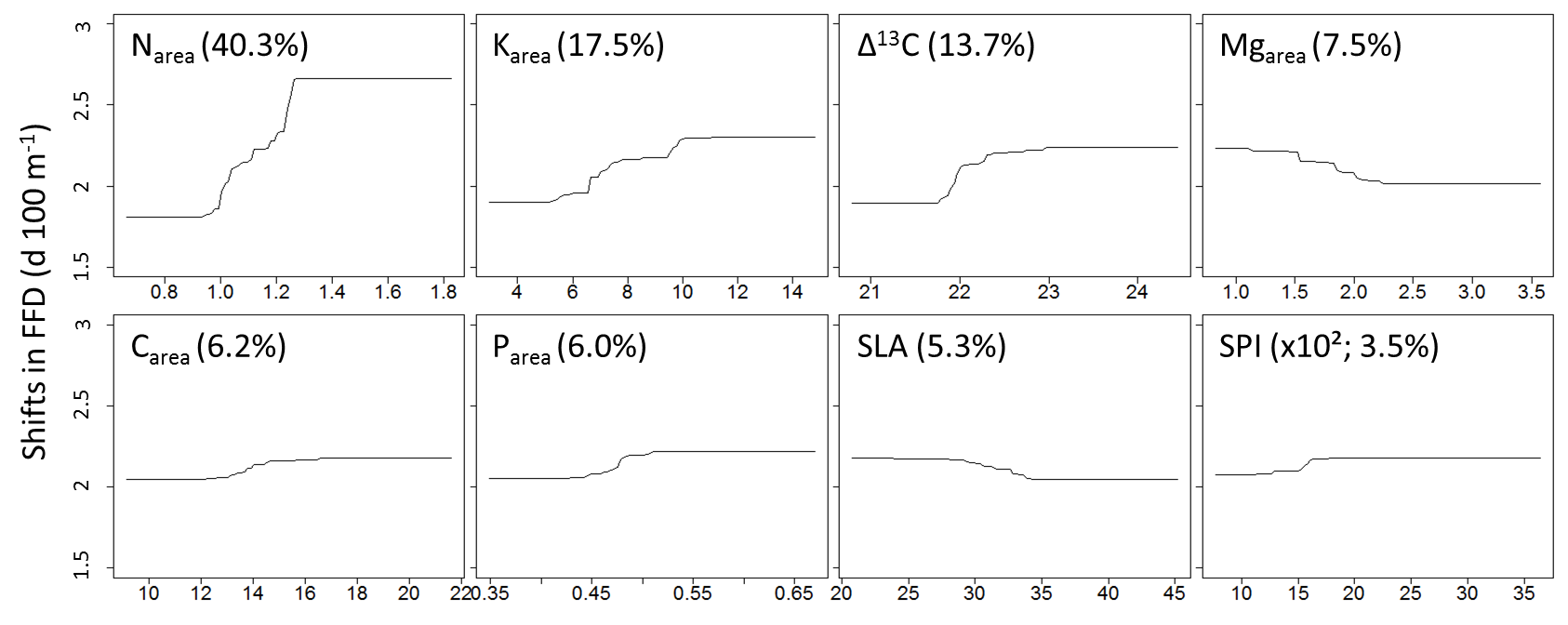


Figure S4: Partial dependency plots of boosted regression trees of relationship between changes in FFD and traits (cv = 0.52). N_area_: Nitrogen per unit leaf area (g m^-2^), K_area_: Potassium per unit leaf area (g m^-2^), Δ^13^C: Discrimination of ^13^C (‰), Mg_area_: Magnesium per unit leaf area (g m^-2^), C_area_: Carbon per unit leaf area (g m^-2^), P_area_: Phosphorus per unit leaf area (g m^-2^), SLA: Specific leaf area (m² kg^-1^) and SPI: Stomatal pore area index (x10²).
