# Supplementary material for: Traits and climate are associated with first flowering day in herbaceous species along elevational gradients
Source: Ecol Evol. 2017 Dec 20;8(2):1147–58. doi: 10.1002/ece3.3720 (PMC5773311; doi:10.1002/ece3.3720)
Supplement: Supplementary file 5 [file ECE3-8-1147-s005.docx]

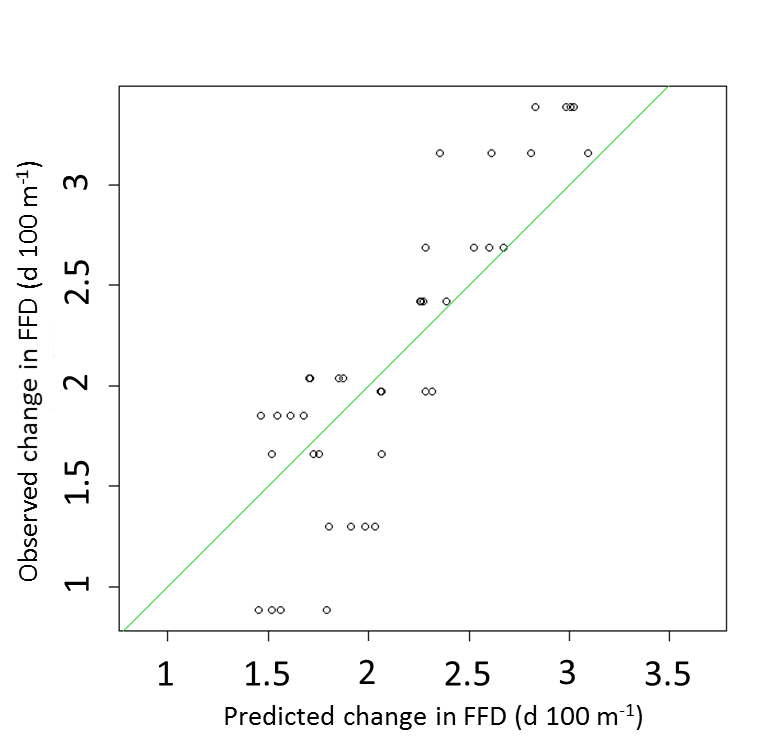


Figure S5: Predicted change of FFD (in days per 100 m increase in elevation) based on booted regression trees plotted against observed FFD (cv = 0.52).
