# Supplementary material for: Traits and climate are associated with first flowering day in herbaceous species along elevational gradients
Source: Ecol Evol. 2017 Dec 20;8(2):1147–58. doi: 10.1002/ece3.3720 (PMC5773311; doi:10.1002/ece3.3720)
Supplement: Supplementary file 6 [file ECE3-8-1147-s006.docx]

Table S1: Mean flowering day (in day of the year) of the selected species on the two elevational gradients (Kramer: Kra and Kreuzeck: Kre) as well as the changes of FFD with elevation and growing degree day per elevation.

|  | Mean flowering day | | | | Days 100 m^-1^ | Growing degree days 100 m^-1^ | | | | |
| --- | --- | --- | --- | --- | --- | --- | --- | --- | --- | --- |
|  | Kra- 2012 | Kre- 2012 | Kra- 2013 | Kre- 2013 |  | Kra- 2012 | Kre- 2012 | Kra- 2013 | Kre- 2013 |  |
| Aposeris foetida (L.) Less. | 143.5 | 149.0 | 146.9 | 158.0 | 2.20 | -16.2 | -9.1 | -8.1 | -1.01 |  |
| Aster bellidiastrum (L.) Scop. | 153.7 | 155.4 | 143.1 | 147.3 | 2.34 | -14.6 | -7.6 | -6.6 | 0.5 |  |
| Buphthalmum salicifolium L. | 188.2 | 192.3 | 193.7 | 194.4 | 2.26 | -17.5 | -10.4 | -9.4 | -2.4 |  |
| Carduus defloratus L. | 185.0 | 187.1 | 186.2 | 193.2 | 3.28 | -8.5 | -1.4 | -0.4 | 6.6 |  |
| Knautia dipsacifolia Kreutzer | 202.2 | 203.6 | 207.4 | 208.3 | 1.29 | -30.5 | -23.4 | -22.4 | -15.4 |  |
| Lotus corniculatus L. | 177.1 | 170.7 | 176.0 | 169.7 | 1.83 | -24.6 | -17.6 | -16.6 | -9.5 |  |
| Mercurialis perennis L. | 129.2 | 132.3 | 119.9 | 122.8 | 0.85 | -14.9 | -7.8 | -6.8 | 0.2 |  |
| Phyteuma orbiculare L. | 169.4 | 175.3 | 176.9 | 183.1 | 3.26 | -8.8 | -1.7 | -0.7 | 6.3 |  |
| Potentilla erecta (L.) Raeusch. | 160.1 | 164.5 | 158.4 | 166.7 | 1.68 | -15.9 | -8.9 | -7.8 | -0.8 |  |
| Trifolium pratense L. | 185.7 | 183.3 | 184.3 | 184.2 | 3.06 | -11.5 | -4.4 | -3.4 | 3.6 |  |
